# Laterality of Facial Expressions of Emotion: Universal and Culture-Specific Influences

**DOI:** 10.1155/2004/786529

**Published:** 2004-06-09

**Authors:** Manas K. Mandal, Nalini Ambady

**Affiliations:** ^1^Indian Institute of Technology–KharagpurIndia; ^2^Tufts UniversityMedfordMAUSA

## Abstract

Recent research indicates that (a) the perception and expression of facial emotion are lateralized to a great extent in the right hemisphere, and, (b) whereas facial expressions of emotion embody universal signals, culture-specific learning moderates the expression and interpretation of these emotions. In the present article, we review the literature on laterality and universality, and propose that, although some components of facial expressions of emotion are governed biologically, others are culturally influenced. We suggest that the left side of the face is more expressive of emotions, is more uninhibited, and displays culture-specific emotional norms. The right side of face, on the other hand, is less susceptible to cultural display norms and exhibits more universal emotional signals.

## 1. Introduction

*"we respond to gestures... in accordance with an elaborate and secret code that is written nowhere, known by none and understood by all"* [120, p. 892].

Human beings rely extensively on nonverbal channels of communication in their day-to-day emotional as well as interpersonal exchanges. The verbal channel, language, is a relatively poor medium for expressing the quality, intensity and nuancing of emotion and affect in different social situations. Nonverbal channels of communication that transmit emotional messages include facial expressions, paralanguage, gestures, gaze, posture, and proximal behavior. Amongst these channels, the face is thought to have primacy in signaling affective information [102]. First, the face is exposed to the full view of others in order to facilitate social interaction. Second, the amount (especially in short period of time) and type of information (e.g., emotional, attitudinal) conveyed by the face are relatively easy to comprehend [59]. It is not surprising, then, that the face has been a central focus of research on the communication of emotion, beginning with the classic work of Darwin [38], and followed up by pioneers in the field such as Tomkins [136], Izard [67], and Ekman [43].

During the past two decades, a great deal of attention has been paid to the issues of the lateralization and universality of facial expressions of emotion. The focus of the lateralization issue has been on the relative role of the two cerebral hemispheres in the understanding, expression and subjective experience of emotion. Primary questions of interest include: (a) Is there a hemispatial advantage in the *identification* of facial expressions of emotion?, (b) To what extent do the two hemifaces differ in the *expression* of positive or negative emotions?, and, (c) Which hemisphere is more activated during the *subjective experience* of different emotions?

The focus of the universality issue has been on the panculturality of recognition and expression of facial emotion. Primary questions of interest that have been examined in the literature include: (a) Are facial emotions recognized similarly across all cultures?, (b) To what extent does culture influence the expression of emotion?, and, (c) Are there any culture-specific emotions?

The purpose of the present article is to review evidence for the laterality and universality of facial expres-

ISSN 0953-4180/04/$17.00 © 2004 – IOS Press and the authors. All rights reserved sions of emotion and to ascertain their possible relationship. To review the evidence, computerized databases (involving search engines like PsychInfo, ScienceDirect, PubMed, Google) were utilized, in addition to examining relevant journals. The selection criteria for studies involved including any that dealt with the functional laterality of facial expressions of emotion, with the major focus being on the recognition and expression of emotion. Studies dealing with the neural pathways in the recognition or the anatomical pathways in the expressions of facial emotions were not included. Experimental as well as clinical evidence was reviewed in order to garner further evidence for the functional laterality of facial expression of emotion. Studies examining the universality and culture-specificity of facial expression of emotion were also included. No attempt was made to determine the efficacy of any particular theoretical position that supported either a universal or a culture-specific perspective. Instead, evidence for both perspectives was examined by focusing on laterality studies.

The impetus behind the examination of the lateralization of emotion was drawn mainly from recent observations that emotion regulation is not restricted to subcortical and limbic structures. A substantial body of research has confirmed the role of the neocortex in the understanding, expression and subjective experience of emotion [18,69]. Interestingly, this work has largely overlooked evidence that facial expression, and judgment of emotions differ widely amongst cultures as a function of emotion type and hedonic valence (but see [83,140]), and the degree of subjective experience of emotion [137].

## 2. Laterality & facial emotions

The study of laterality provides a window into the understanding of behavioral and neural processes [60], especially those pertaining to emotion [26]. The term “laterality” indicates anatomic and functional differences between the two halves of the brain [143]. Anatomical differences are referred to as biological asymmetry (like the relative size, shape of the two hemispheres, brain-ventricle ratio, skull size, etc.) and functional differences are referred to as behavioral asymmetry. Behavioral asymmetry is determined either via central (e.g., split-visual-field, dichotic listening, dichhaptic techniques, etc.) or peripheral (e.g., facedness, handedness, footedness, etc.) measures. The central measures directly assess perceptual processes in the two hemispheres. Peripheral measures indirectly ascertain the role of two hemispheres in motor expressions.

Two approaches are generally taken in examining hemispheric involvement in affective processing: experimental approaches, in which normal functions of the brain are studied usually with participants whose brains are intact (although, at times brain-damaged patients are also tested in experimental studies); and clinical approaches, the goal of which is to examine the degree and magnitude of functional impairment following localized brain damage [128]. Both approaches deal with the hemispheric involvement in affective functioning, although observations derived from these two approaches are not directly comparable.

## 3. Lateralization in the identification of facial emotion: Experimental and clinical evidence

Table 1 presents the experimental evidence for perceptual asymmetry of facial expressions of emotion. This evidence, in general, suggests that the left visual-field (a contralateral function of the right hemisphere) is superior in the identification of facial expressions of emotion, especially negative emotions. Most of these studies utilize a split visual-field technique in which facial expressions are presented to two visual-fields (with the observer’s eyes fixed on a central point) for 180 ms or less. This method ensures that left and right visual images initially reach the contralateral hemispheres. The left visual-field superiority for facial emotion identification has been found to be robust across studies that used photographs, cartoons, or schematic drawings. Left visual-field superiority has also been found when stimulus photographs are presented under neutral or free-viewing conditions.

These experimental findings have been substantiated by clinical evidence. For example, studies on clinical samples reveal that there is strong evidence that right-hemisphere damage relative to left-hemisphere damage impairs the perception of emotional expressions displayed via the face [20,35,77,80,83,88], the comprehension of emotional prosody [111], the judgment of emotion-laden lexicons [127], the understanding of an emotional tone of voice [61], and the appreciation of humorous stimuli [10]. Moreover, right hemisphere-damaged patients, in comparison to left hemisphere-damaged patients, also have difficulty in naming the emotions conveyed by different facial expressions [23, 36,53].

Table 1
Studies on Visual-field Asymmetry during Perception of Emotion

| Authors | General observations |
|---|---|
| Campbell,1982<br>Davidson and Fox,1988<br>Gianotti, 1972<br>Silberman and Weingartner, 1986 | Right hemisphere involvement in perception of negative emotion and the left hemisphere involvement in perception of positive emotions were documented |
| Ley and Bryden, 1979 | Left visual-field superiority for recognition of emotion was found. The effect was more for negative than positive emotions |
| Butchel et al., 1978<br>Mc Keever and Dixon, 1981 Natale et al., 1983 | Left visual-field (right hemisphere) advantage was found for the processing of all emotions |
| Mandal and Singh, 1990<br>Schweinberger et al., 2003<br>Strauss and Moscovitch, 1981 | Left visual-field advantage (a right hemispheric function) was found for the perception of emotion-laden facial photographs |
| Hoptman and Levy, 1988 | Right hemisphere superiority was found for the perception of facial emotions |
| Patterson and Bradshaw, 1975 | Emotional judgment effect may be due to a general right hemispheric superiority in processing face |
| Strauss and Moscovitch, 1981 | Faster response time in the left visual-field when expressions were the same, but little difference when expressions were different |
| Asthana and Mandal, 2001 | A left visual-field advantage in the perception of sad emotion and no lateral advantage in the perception of happy expression were observed |
| Magnussen et al., 1994 | Both hemispheres contribute to the perceptual analysis of emotional signal depending upon the sign and strength of the emotion expressed |
| Bo d et al., 1989<br>Heller, 1990; Moreno et al., 1990 | Left hemispace bias for the perception of facial emotion in free-field presentation |
| Borod et al., 1989<br><br>Reuter-Lorenz et al., 1983 | A left visual-field advantage for perceiving negative emotions and a right visual-field advantage for perceiving positive emotions |
| Reuter-Lorenz and Davidson, 1981 | Left hemisphere advantage was greater for perception of positive and right hemisphere advantage was greater for perception of negative emotions |
| Jansari et al., 2000 | Negative emotions were perceived better when presented to the left of neutral face and positive emotions were perceived better when presented to the right of neutral face |
| Vingerhoets et al., 2003 | Greater right hemispheric blood flow velocity with attention to affective prosody |

## 4. Lateralization in the expression of facial emotion: Experimental and clinical evidence

In expression studies, actors are required to produce facial expressions in posed or spontaneous conditions. In these studies, facial composites are prepared by cutting the original and mirror-reversed prints of each photograph along the vertical midline and then joining the appropriate sides (e.g. [118]). The left-left (LL) composite is thus produced by joining the left hemiface of normal orientation and its mirror image. Similarly, the right-right (RR) facial composite is prepared by assembling the right hemiface of normal orientation and its mirror image. Observers are asked to rate these composite photographs in terms of intensity of expression (see also [84]).

Findings from these experiments suggest a left hemifacial bias for both posed and spontaneous expressions of emotion (See Table 2). A left hemifacial bias in emotion expression presupposes the right hemispheric involvement because of the crossed cortical dominance by the way of contralateral fibre connections. Meta-analyses also show compelling evidence for a left hemiface bias, especially during negative emotion expressions; the effect size is less pronounced for a right hemiface bias for positive emotion expressions (see [16,84, 131]).

These findings have been substantiated with clinical

Table 2
Facial asymmetry during expression of emotion

| Authors | General findings |
|---|---|
| Sackeim and Gur, 1978 | Greater intensity on the left side of the face except happy |
| Campbell, 1979<br>Kowner, 1995 | Left-sided facial asymmetry for positive emotions |
| Kop et al., 1991<br>Schwartz et al., 1979 Sirota and Schwartz, 1982 Strauss and Kaplan, 1980 | No facial asymmetry for either positive or negative emotions |
| Borod and Caron, 1980 | No facial asymmetry for positive and left-sided facial asymmetry for negative emotions |
| Cacioppo and Petty, 1981 | No facial asymmetry for negative emotions |
| Ekman et al., 1981 | No facial asymmetry for negative emotions |
| Heller and Levy, 1981<br>Rinn et al., 1982 | Left-sided facial asymmetry for positive emotions |
| Ladavas, 1982 | Left-sided facial asymmetry for adults but not for young participants |
| Asthana and Mandal, 1998<br>Baribeau et al., 1987<br>Borod et al., 1988<br>Borod et al., 1990<br>Dopson et al., 1984<br>Mandal et al., 1993<br>Moscovitch and Olds, 1982<br>Moreno et al., 1990 | Left-sided facial asymmetry for either positive or negative emotions |
| Sackeim et al. 1984 | No consistent facial asymmetry for any region |
| Hager and Ekman, 1985<br>Monserrat, 1984 | Left-sided facial asymmetry for positive and no asymmetry for negative emotions |
| Mandal et al., 2001<br>Mandal and Singh, 1990<br>Wemple et al., 1986 | Left-sided facial asymmetry for negative emotions |
| Sackeim and Grega, 1987<br>Schiff and Lamon, 1989 | Left-sided facial asymmetry for negative and no asymmetry for positive emotions |
| Brockmeier and Ulrich, 1993<br>Schiff and MacDonald, 1990 | Left-sided facial asymmetry for negative and right-sided facial asymmetry for positive emotions |
| Mandal et al., 1995 | Right sided asymmetry for intense emotion expression |
| Yecker et al., 1999 | Greater right-sided facial asymmetry for approach and left-sided for withdrawal expressions |
| Nicholls et al., 2002 | Left sided facial asymmetry for emotion expressions |
| Indersmitten and Gur, 2003 | Emotions are expressed more intensely on the left except anger that is more intensely expressed on the right face |

evidence examining the ability of patients with focal brain damage to communicate emotions via the face. In a review of these studies, Borod [12] found that right hemisphere-damaged patients were significantly more impaired than left hemisphere-damaged patients for posed [20,25,68] and spontaneous [29,90,110] facial expressions of emotion. Based on this evidence, Borod [11,13] conceptualized two hypotheses with regard to hemispheric involvement in emotional behavior: (a) a right-hemisphere hypothesis, and, (b) a valence hypothesis. The first hypothesis proposes that the right hemisphere is dominant for all kinds of emotions (see also [28,60]). The second hypothesis assumes that the right hemisphere is specialized for negative emotions and the left hemisphere is specialized for positive emotions (see also [129]). A variant of this hypothesis assumes that the two hemispheres are differentially specialized for the expression and experience of emotion as a function of valence but not for the perception of emotion. According to this view, the right hemisphere is specialized for the perception of emotion irrespective of valence (see also [26,39]).

In sum, the evidence in favor of right-hemisphere involvement in emotion perception and expression is overwhelming [14,72]. Bowers, Bauer, and Heilman [24] reviewed evidence from neuropsychological studies and concluded that the right hemisphere contains a "vocabulary" of nonverbal affective signals (facial expressions, prosody, and gestures), supporting the notion of a general processor (rather than specific processor for particular facial expressions, prosody, etc.) for emotion processing in normal subjects [21].

But a role for the left hemisphere in the earliest level of processing of facial emotions has not been ruled out. Rinn [108] speculated that "(the) right hemisphere lateralization for emotion may actually be due to a left superiority for the inhibition of emotion" (p. 73). Further, social perception depends on the ability to dissociate signals from social and nonsocial situations. This function may be mediated primarily by the left hemisphere. Social situations embody both perceptual as well as conceptual cues. Conceptual information has been found to be mediated by the left hemisphere, and perceptual information has been found to be mediated by the right hemisphere [147].

The involvement of the right hemisphere in positive affect states is also somewhat controversial. Some investigators have demonstrated a bilateral advantage for the processing of positive emotion whereas others have documented a left-hemispheric advantage for the processing of such emotions [11]. Researchers have also speculated about a distinction in terms of motoric direction as well as approach-withdrawal [41] in addition to a positive-negative distinction within the emotion categories. The roles of hedonic valence and motoric direction were tested in a recent study on brain-damaged patients [80]. Right hemisphere-damaged patients had specific deficits relative to left hemisphere-damaged patients in processing negative and withdrawal emotions; there was a non-significant group difference for positive/approach emotions.

The issue of posed vs. spontaneous expressions has generated some controversy. Posed expressions are produced voluntarily whereas spontaneous expressions are produced automatically in response to an affective situation. Anatomical evidence suggests different anatomical involvement during these two situations; for example, neocortical structures have been implicated for posed expression and subcortical structures for spontaneous expressions [**?**,95]. Though many experimental findings suggest a left hemifacial bias for both posed and spontaneous expressions of emotion, some studies do not report a hemifacial bias for spontaneous expressions (for example [31,47,117,130].

Finally, facial musculature is not solely controlled by the contralateral mechanism of the brain (the right hemisphere – the left side of the face). It has been observed that the muscles in the lower region of the face are contralaterally innervated by the fibre projection of the two cerebral hemispheres [20,108]; however, the facial nerves originating in the right hemisphere are distributed uncrossed to the upper region of the face. Supporting these obersvations, a recent study found that the right upper face is more expressive than the left upper face [7].

## 5. Universality and culture-specificity

Having examined the issues related to laterality and emotion, we now turn to the question about the innateness versus the cultural specificity of facial expressions of emotion. Neurophysiologists, cultural anthropologists, and psychologists have been addressing this issue for several decades. Beginning with the influential early work by Darwin [37,38] that favored universality, researchers in psychology have spent decades examining the issues of the universality and cultural specificity of the expression, experience, and judgment of emotion. Extreme positions taken by early theorists have gradually given way to recent theoretical models with an interactionist perspective that suggest a role for both universality and cultural specificity (e.g. [43,55,89,93, 94,110,122]). Proponents of this view consider emotion as a composite of several subsystems that include: (a) antecedent events, (b) emotional experience, (c) appraisal, (d) physiological change, (e) change in action readiness, (f) behavior, (g) change in cognitive functioning and beliefs, and, (h) regulatory processes [56, 74,104,121]. Each subsystem of a modal emotion may vary as a result of variation in culture and, therefore, it is essential that universality has to be examined for each of these subsystems of an emotion before generalizations can be made.

Ekman [43,44] suggested that facial emotions are expressed in a universally similar manner. He maintained that primary emotions are expressed by a combination of facial muscular movements that are neurally connected. Six primary emotions were identified with distinct facial muscle combinations during expressions happiness, sadness, fear, anger, surprise, and disgust. Two lines of evidence are available to support Ekman's view, dealing with (a) the decoding (understanding) of facial emotion, and, (b) the encoding (expression) of facial emotion. Decoding studies require observers of different cultures to judge the facial expression of primary emotions, namely, happiness, sadness, fear, anger, disgust, and surprise. Facial expressions of emotion do seem to be recognized panculturally in Western and non-Western cultures [15,43,46,49,67,91,101] as well as in literate and preliterate populations [22,45,49, 132].

In comparison to decoding studies, attempts made to test universality in the expressions of primary emotions have been relatively less frequent. Encoding studies require that the subjects of different cultures express basic facial emotions. These emotions are produced either by imagining affective situations, or by following instructions to move facial muscles in a definite pattern. Some data suggest that facial emotional expressions are displayed by all cultures as long as socially learned display rules do not interfere [48].

Russell [114], on the other hand, has argued that the recognition of facial expressions of emotion depends to a large extent on the sender's and receiver's language and culture (see also [50]). Reviewing the cross-cultural evidence for the recognition of facial expression of emotions, Russell concluded that "facial expressions and emotion labels are probably associated, but the association may vary with cultures and is loose enough to be consistent with various alternative accounts" [113, p. 102].

In examining the classic data on emotion recognition along with newer cross-cultural evidence, a recent meta-analysis has provided evidence for an "ingroup advantage" in emotion recognition [50,51]. That is, emotion recognition is more accurate when members of the same cultural group that express the emotions also make the judgments. This ingroup advantage has been seen across a range of experimental methods and nonverbal channels of communication, as well as across each of the basic emotions, both positive and negative. Further, the ingroup advantage has been found when examining only balanced studies, in which members of every cultural group in the study judged emotions expressed by members of every other group. Such balanced studies control for possible differences in the main effects of emotional expression and recognition ability across cultures, while examining the impact of cultural match or mismatch on communication accuracy in the form of an interaction effect. Thus, there is strong evidence for an ingroup advantage in emotional decoding even after controlling for absolute differences across cultures.

The universality thesis argues that facial expressions of emotion are uniformly understood, expressed or experienced across cultures (for example [44]). Opponents of this view (cultural relativism) suggest that, despite universality, culture plays a major role in the understanding and expression of facial emotion and that emotion expressions are a natural outgrowth of cultural learning [8,104]. Their argument is grounded in ecological demand, ethnic variation, social construction of the self, and cultural practices. These two extreme views rarely acknowledge the fact that biological and social signals may be accommodated in an opposite but complementary manner in facial emotion research. To understand this interface, contributions from lateralization research can be utilized that suggest an asymmetry of both the expression and the perception of facial expression. We suggest that linking the judgment of facial expression with findings on laterality might provide one way to disentangle innate and culture-specific effects. The laterality literature suggests that facial expressions of emotion, though distinct and universal, are not uniformly displayed or consensually judged. Asymmetry in facial expressions of emotion may be produced as a result of a variety of factors such as neurobiological constraints as well as by cultural factors [140].

The bulk of the literature on laterality and facial emotion reviewed earlier in this paper suggests that (a) facial expressions of emotion are better perceived in the left than in the right visual-field (a right-hemisphere dominance) of the perceiver, and, (b) the left side of the face, in comparison to the right, displays emotion in a more pronounced manner (a right-hemisphere dominance). With regard to the first proposition, that emotion judgments are governed by right-hemisphere dominance, some authors argue that the left visual-field dominance is found generally for face recognition rather than specifically for recognition of facial emotion. Neuropsychological research has established that the two processes (face recognition and recognition of facial emotion) are separate. Prosopagnosic patients (who do not recognize familiar faces following damage to the visual system) retain the ability to recognize facial expressions of emotion [54]. Studies with normal subjects have also documented a difference in performance on face-identity judgment tasks and face-emotion judgment tasks [133]. The second proposition, that emotional displays are right-hemisphere dominant, has also been criticized with the argument that the left hemifacial dominance in emotion production may be a function of non-emotional peripheral factors. For example, if the two hemifaces differ in the degree of muscular activity, the hemiface with greater mobility might be perceived as more emotionally expressive. But the role of the peripheral factors in the production and judgment of neutral expression in comparison to emotion expression is significantly reduced. Yet the resting left hemiface is judged either more happy [135] or miserable [32] than the right hemiface. In one study,

Mandal, Asthana, Madan, and Pandey [81] examined the asymmetrical nature of the resting (neutral) face by preparing hemifacial composites, left-left, right-right, and a normal facial orientation. The left-side facial composites were found to be more emotional than the right-side or normal facial orientations of neutral expressions.

These two propositions suggest that in face-to-face interactions the dominant side of the face does not fall into the dominant visual-field of the perceiver. Other researchers have also tested similar face-to-face situations (for example [118]) and have examined the impact of lateralization of facial expression on the attribution of personality [71], and facial attractiveness [146]. For instance, Asthana and Mandal [4] tested a speculation that the mirror-reversal of a facial expression would be perceived as more intense in comparison to its normal orientation because in such a case the hemiface (left), dominant for emotion expression, will be processed by the side of a hemisphere (right) that determines judgment about a whole face in a free-viewing condition. As predicted, expressions in the mirror-reversed orientation were perceived as more intense than those in the normal orientation. Based on these findings, Asthana & Mandal suggested two possibilities as to why the emotional tone in the left hemiface eludes day-to-day notice. First, "we learn in the course of human development to civilize intense emotional expressions . . . by the side of the face (right) which is more under the voluntary motor control of the [left] cerebral hemisphere." Alternatively, "we evolve a strategy in the course of civilization to process the emotionally pronounced side of the face (left) by the hemisphere side (left) that largely mediates cognitive ability. . . (p. 117)". These speculations were, however, made based on evidence drawn under laboratory conditions. Further studies are needed to substantiate these views with more recent methodologies involving visual scan paths.

Taken together, these studies suggest that although some components of facial emotion are more biologically governed, others are moderated to great extent by cultural experience. Undeniably, therefore, these two components develop an interface to facilitate social communication. Relatively less theoretical energy has been expended to examine the reciprocal nature of the biological and cultural components of facial expressions of emotion. But the literature does provide evidence upon which such a theory can be built. For example, neuropsychological research has shown that the two sides of the human face are not equally expressive, and that 'asymmetrical facial expressions have some relationship to the functional asymmetry of the brain' [140]. The right side of a face (controlled by the left hemisphere that primarily mediates cognitive behavior) offers socially appropriate clues whereas its left side (controlled by the right hemisphere that primarily mediates emotional processes) divulges hidden personalized feelings (see for example [4]). This proposition was originally made by Wolff [144] who suggested that the left side of the face expresses more personalized, hidden and unconscious content while the right side of face reveals more social, explicit and conscious content of personality (see also [118]). Support for the differential hemispheric involvement in facial expression is drawn from both clinical as well as experimental studies. Neuroanatomically, the left side of the face is motorically governed by the right side of the brain (via contralateral fibre connections), which is relatively specialized for processing of emotions; the left side of the brain, which is relatively specialized for cognitive processing, governs the right side of face. Buck [28] and Tucker [138] also suggest that the right hemisphere is associated with emotional processes and the left hemisphere with the control (i.e., facilitation, inhibition) of these processes as per the socially approved rules.

Given such evidence, one may argue that the left in comparison to the right side of the face should be more emotional, uninhibited and culture-specific. Expressions of the right side of face, on the other hand, should be pancultural, and should exhibit emotional expressions in accordance with universal norms. Universal expressions are expected to be displayed by the right side of face due to greater voluntary motor control which is contralaterally connected with the relatively less emotional side of the cerebral hemisphere (left) for the facility of social interaction [4].

Two recent studies were conducted that examined this claim. In one study, hemifacial composite photos (left-left, right-right, and normal orientation) of three cultures - Japanese, Oriental Indian, and North American - displaying six emotions happy, sad, fear, anger, surprise, disgust, and a neutral state - were judged by Indian observers for distinctiveness of expression. The findings suggest that facial emotions were displayed in a universal manner; however, there was a subtle difference in the hemifacial involvement of expression. Although North Americans showed left hemifacial bias for all emotions, Japanese showed a right hemifacial bias for positive and left hemifacial bias for negative emotions. Negative emotional expressions were least distinctly identifiable in Japanese faces, followed by Indian and North American faces [85].

In another study, the in-group advantage in emotion judgment was examined as a function of the hemifacial differences in expressions. Participants from the USA, India, and Japan judged facial expressions from all three cultures in a balanced design. The right-right facial composites, in comparison to the left-left composites, yielded more cross-cultural agreement. More specifically, the in-group advantage was greater for the left-left than the right-right composites. These findings suggest that the left side of the face has an expressive style that is more culture-specific and less universal [52].

These studies do not conclusively prove that the two hemifaces differ as a function of the differential influence of biology and culture. A host of factors may influence facial asymmetry including anatomical, neurological, psychological, pathological, and socio-cultural factors [140]. More studies are needed to examine the theory proposed above that control for variables such as hemiface size, developmental changes, age, sex, etc. Such studies will pave the way towards the development of cross-cultural neuropsychology [2] in the communication and experience of emotion. Cross-cultural neuropsychology is an emerging discipline that examines behavioral neuroscience within a cultural context. Behavioral neuroscience explores the biological bases of behavior by drawing from the fields of neurospsychology, neurophysiology, psychopharmacology, neuroanatomy and neuroendocrinology. Cultural psychology, on the other hand, understands behavior from the interdisciplinary perspectives of anthropology, behavioral ecology and social and developmental psychology. The interest in examining cultural issues in cognitive neuropsychological performance has generated a great deal of research, for example, the influence of language [1,139], literacy [3], socio-educational factors [103], and cultural norms [27]; for details see Ardila [4]. Very few attempts have been made, nevertheless, to examine the relationship between culture and the neuropsychology of emotion. We suggest that examining laterality effects in cross-cultural studies of facial expressions of emotion will advance our understanding of both the communication and the construction of emotion.

## Acknowledgement

Preparation of this article was supported by a Fulbright Visiting Lecturer Grant to MKM at Harvard University, USA to the first author and a NSF Presidential Early Career Award to the second author. Correspondence concerning this article may be addressed to Manas K. Mandal, Department of Humanities and Social Sciences, Indian Institute of Technology – Kharagpur – 721302, India. The authors thankfully acknowledge the help of Hari O. Sharma in preparation of this article.
